# The combined effect of USP7 inhibitors and PARP inhibitors in hormone-sensitive and castration-resistant prostate cancer cells

**DOI:** 10.18632/oncotarget.16463

**Published:** 2017-03-22

**Authors:** Francesco Morra, Francesco Merolla, Virginia Napolitano, Gennaro Ilardi, Caterina Miro, Simona Paladino, Stefania Staibano, Aniello Cerrato, Angela Celetti

**Affiliations:** ^1^ Institute for Experimental Endocrinology and Oncology, Research National Council, Naples, Italy; ^2^ Department of Advanced Biomedical Sciences, University “Federico II”, Naples, Italy; ^3^ Department of Medicine and Health Sciences “V. Tiberio”, University of Molise, Campobasso, Italy; ^4^ Department of Molecular Medicine and Medical Biotechnology, University “Federico II”, Naples, Italy

**Keywords:** CCDC6, USP7, ARFL and V7, P5091, olaparib

## Abstract

**Purpose of the study:**

Reduced levels of the tumor suppressor protein CCDC6 sensitize cancer cells to the treatment with PARP-inhibitors. The turnover of CCDC6 protein is regulated by the de-ubiquitinase USP7, which also controls the androgen receptor (AR) stability. Here, we correlated the expression levels of CCDC6 and USP7 proteins in primary prostate cancers (PC). Moreover, we tested the efficacy of the USP7 inhibitors, in combination with PARP-inhibitors as a novel therapeutic option in advanced prostate cancer.

**Experimental techniques:**

PC cells were exposed to USP7 inhibitor, P5091, together with cycloheximide, to investigate the turnover of the USP7 substrates, AR and CCDC6. As outcome of the AR downregulation, transcription targets of AR and its variant V7 were examined by qPCR. As a result of CCDC6 degradation, the induction of PARP inhibitors sensitivity was evaluated by analyzing PC cells viability and foci formation. We scored and correlated CCDC6 and USP7 expression levels in a prostate cancer tissue microarray (TMA).

**Results:**

P5091 accelerated the degradation of AR and V7 isoform affecting PSA, UBE2C, CDC20 transcription and PC cells proliferation. Moreover, P5091 accelerated the degradation of CCDC6 sensitizing the cells to PARP-inhibitors, that acted sinergistically with genotoxic agents. The immunohistochemical analysis of both CCDC6 and USP7 proteins exhibited significant correlation for the intensity of staining (*p* ≤ 0.05).

**Data interpretation:**

Thus, CCDC6 and USP7 represent predictive markers for the combined treatment of the USP7-inhibitors and PARP-inhibitors in advanced prostate cancer.

## INTRODUCTION

Prostate cancer (PC) is the most common cancer in male and is among the three leading causes of cancer death in men in the United States [1] and in Europe [2]. The activation of androgen receptor (AR) is crucial for PC growth at all stages of the disease and the androgen receptor (AR) signaling is the principal target for prostate cancer treatment [3–5]. However, androgen-deprivation therapies cannot completely abolish AR signaling and most prostate cancers become eventually castration-resistant prostate cancer (CRPC), because of the occurence of AR gene point mutations or truncation [6].

Recently, it has been demonstrated that the stability of the androgen receptor in prostate cancer cells is regulated by the de-ubiquitinase USP7, also known as herpesvirus-associated ubiquitin-specific protease [7]. USP7 has been identified as a co-regulator and an interactor of androgen receptor (AR) in an androgen-dependent manner. Moreover, USP7 mediates the receptor deubiquitination and allows the AR binding to the chromatin for the transcription of specific genes that promote cell proliferation [8]. Thus, the expression of USP7 has been directly correlated to prostate cancer aggressiveness [9] and has been considered a possible target of therapy, in this tumor type [10]. USP7 de-ubiquitinase has varied substrates such as the transcription factor FOXO4, the TP53-regulator MDM2, the tumor suppressors PTEN and CCDC6, besides the AR [11–16].

In particular, CCDC6 gene product is involved in DNA damage and repair processes [17–19]. In primary tumors, the impaired function of CCDC6 protein has been ascribed to CCDC6 gene rearrangements or CCDC6 somatic mutations [20–21]. Moreover, altered levels of CCDC6 protein in cancer cells seem to depend on the altered turnover of CCDC6 protein regulated by the de-ubiquitinase USP7 [16].

Recently, we have reported that low levels of CCDC6 associates with an impariment of Homologous Recombination (HR) mechanisms affecting cells behaviour and cells sensitivity to PARP inhibitors treatment in lung and colon cancer models [16, 22]. Moreover, we have reported a combined effect of the inhibitors of USP7 and PARP enzymes in the treatment of lung neuroendocrine tumor cells expressing USP7 and CCDC6 proteins [23].

Emerging data suggest that PARP inhibition is a potentially important strategy for managing a significant subset of prostate tumors [24–28].

In this study we have investigated whether the pharmacological inhibition of USP7, by impairing the stability of AR, is able to weaken the AR-dependent proliferation of prostate cancer cells. Moreover, in these cells, we have investigated whether the inhibition of the deubiquitinase USP7, by lowering the CCDC6 levels and impairing the homologous recombination (HR) processes may increase the prostate cancer cells sensitivity to PARP inhibitors.

Interestingly, we have detected targettable levels of USP7 and CCDC6 in 68% of analysed tumors, in a Tissue Micro Array (TMA) of 28 primary prostate tumors. Thus, our data suggest that the USP7 inhibition represents a compelling therapeutic strategy for hormone-sensitive and androgen-resistant prostate tumors. Moreover, the inhibition of USP7 enzyme could be considered an ideal treatment, in combination with the PARP inhibitors, in both hormone-sensitive and androgen-resistant prostate tumors that express USP7 and CCDC6.

## RESULTS

### Pharmacological inhibition of USP7 affects prostate cancer cell proliferation

**Figure 1:**
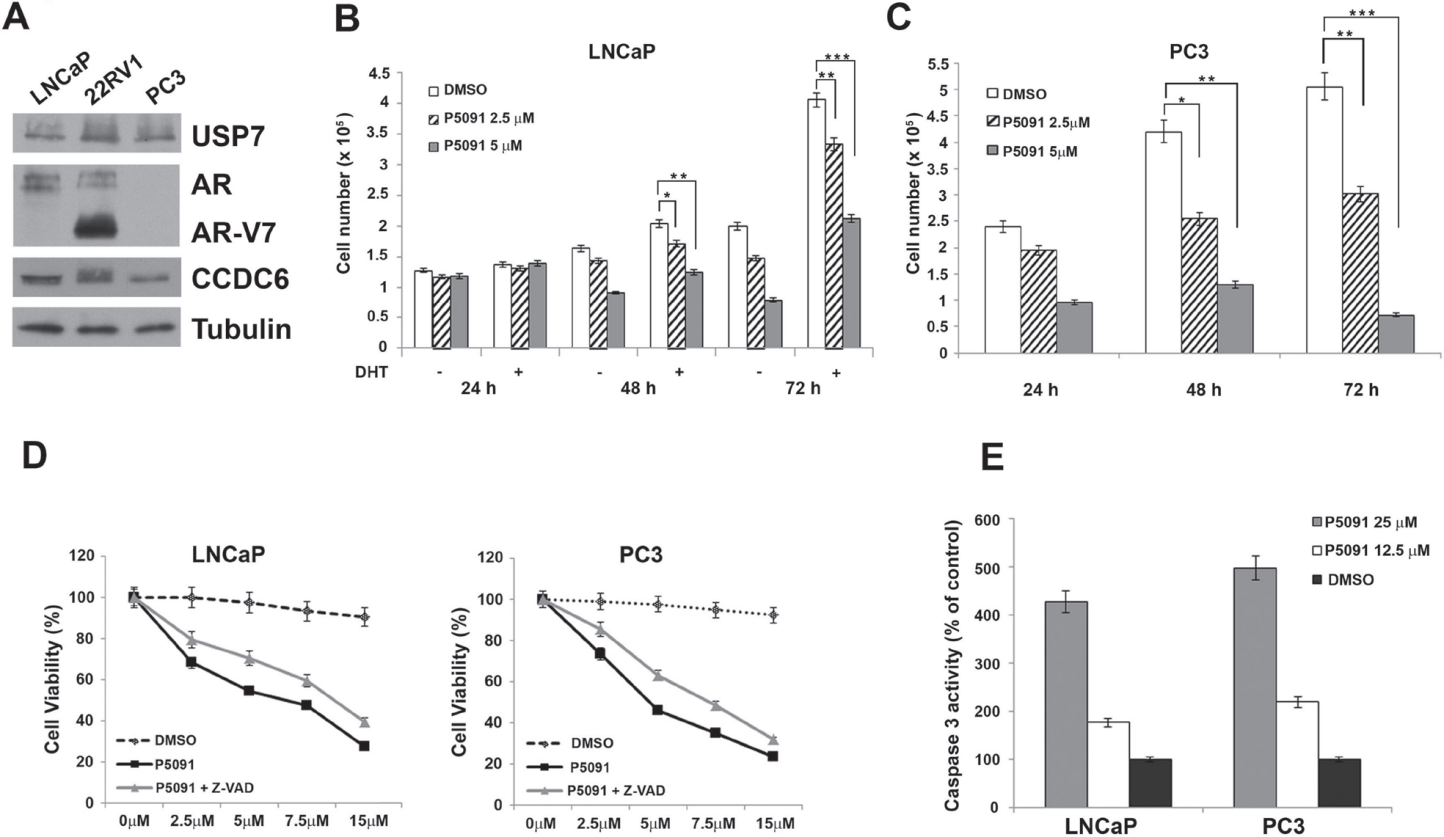
Pharmacological inhibition of USP7 affects prostate cancer cell proliferation (**A**) Immunoblot analysis of USP7, CCDC6, AR and ARV7 isoform in human LNCaP, 22RV1 and PC3 prostate cancer cell lines. Antitubulin is shown as loading control. (**B**) LNCaP cells were treated with vehicle or different concentrations of P5091, as indicated, and cells were counted at the indicated times, in the presence or absence of DHT (10 nM). (**C**) PC3 cells were treated with vehicle or different concentration of P5091, as indicated, and cells were counted at the indicated times. In B and C the values are the mean +/− SD of three independent experiments. (**D**) USP7 inhibitors P5091 shows dose-dependent cytotoxic effect in prostate cancer cell lines. Cells were seeded in 96-well plates and 24 h later exposed to vehicle or P5091 at the indicated doses, in presence or absence of Z-VAD-FMK (20 μM), for 144 h and analysed for viability using a modifeid 3-(4,5-dimethylthiazole-2-yl)-2-5-diphenyltetrazolium bromide assay. CellTiter 96 Aqueous One Solution assay (Promega), as 50% inhibitory concentration (IC50) values. The value are presented as mean standard deviation of three independent experiments. Surviving fraction of LNCaP and PC3 cells are shown. (**E**) Caspase 3 activity was evaluated in LNCaP and PC3 cells treated or not treated with P5091 for 24 h, as indicated. The plotted values represent the mean +/− s.e.m. of three independent experiments.

The pharmacological inhibition of USP7 has shown antitumor properties in several tumor types, including multiple myeloma [29], neuroblastoma [30], colon cancer [31], and lung neuroendocrine tumors [23]. Although the mechanisms leading to the antitumor effect of USP7 inhibitors need to be clarified, the efficacy of USP7 inhibitors can be tested in more tumor types, including prostate carcinoma. The hormone-sensitive LNCaP prostate cancer cell lines, that show appreciable levels of USP7 (Figure 1A), were treated with various concentrations of the USP7 inhibitor P5091 or vehicle, with or without DHT stimulation. The cells were counted at different times (24, 48 and 72 hours). The P5091 treatment affected the LNCaP cell number, particularly in presence of DHT, suggesting a key role of USP7 in the growth of hormone-sensitive prostate cancer cells (Figure 1B). However, the P5091 treatment also showed a similar effect in PC3 cells that are negative for the AR expression (Figure 1C).

Both cell types (LNCaP and PC3) showed an increase in the number of apoptotic cells upon USP7 inhibitors treatment, as revealed by different assays. The Z-VAD-FMK pan-caspase inhibitor interfered with the P5091-induced citotoxicity in hormone sensitive and in androgen-independent prostate cancer cells (Figure 1D); moreover, the caspase 3 was activated upon P5091 treatment in both the cell lines (Figure 1E), suggesting overall that the reduced number of cells induced by P5091 treatment is due to apoptosis mediated, at least in part, by caspases.

### The USP7 inhibitors show antiproiferative effects in the androgen resistant 22Rv1 prostate cancer cells

Androgen-deprivation therapy is the most widely used treatment for advanced prostate cancer. During prostate cancer progression, androgen-deprivation therapy is no longer effective, resulting in castration resistant prostate cancer (CRPC) in which the AR signaling is reactivated upon AR gene amplification, mutations, or truncations.

The 22Rv1 cells represent an ideal *in vitro* model of the transition between hormone-sensitive cells and castration resistant prostate cancer cells. These cells express both the AR full lenght and also the ARV7 splice variant, whose activity is ligand-independent (Figure 1A) [32–35].

**Figure 2:**
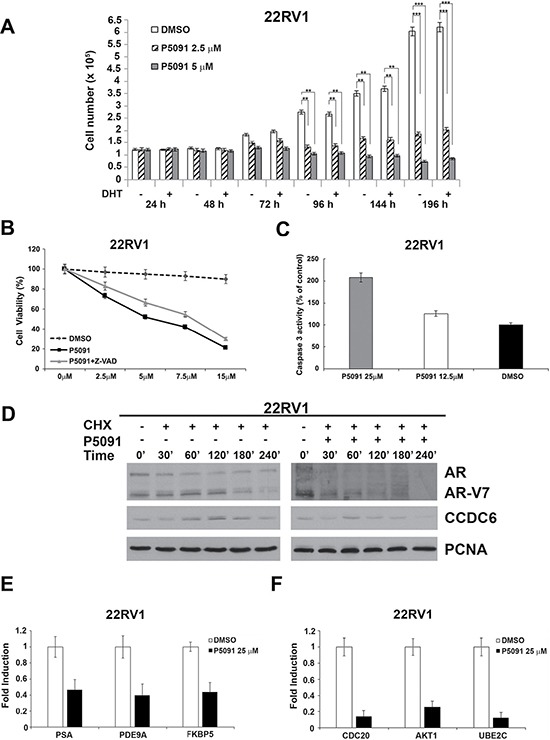
The USP7 inhibitor P5091 shows antiproliferative effects, affects CCDC6, AR and V7-isoform half lives and impairs androgen-responsive genes expression in 22Rv1 cells (**A**) 22Rv1 cells were treated with vehicle or different concentrations of P5091, as indicated, and cells were counted at the indicated times, in the presence or absence of DHT (10 nM). The values are the mean +/− SD of three independent experiments. (**B**) USP7 inhibitors P5091 shows dose-dependent cytotoxic effect in prostate cancer cells. Cells were seeded in 96-well plates and 24 h later exposed to vehicle or P5091 at the indicated doses, in presence or absence of Z-VAD-FMK (20 μM), for 144 h and analysed for viability using a modifeid 3-(4,5-dimethylthiazole-2-yl)-2-5-diphenyltetrazolium bromide assay. CellTiter 96 Aqueous One Solution assay (Promega), as 50% inhibitory concentration (IC50) values. The value are presented as mean standard deviation of three independent experiments. Surviving fraction of 22Rv1 cells is shown. (**C**) Caspase 3 activity was evaluated in 22Rv1 cells treated or not treated with P5091 for 24 h, as indicated. The plotted values represent the mean +/− s.e.m. of three independent experiments. (**D**) 22Rv1 cells were pretreated with either vehicle or P5091 (6 μM, IC50 in 22Rv1 cells), for 4 h, followed by the addition of cycloheximide (CHX) at 50 μg/ml for the indicated times. Total proteins lysates were subjected to immunoblot analysis using anti-CCDC6, anti-AR (able to detect the full lenght and the V7 isoform) or anti-PCNA antibodies. (**E**) Expression of AR-target genes levels in 22Rv1 cells was determined by qPCR, following vehicle or P5091 treatment (25 μM) for 24 h, and normalized against expression of GAPDH. (**F**) Expression of ARV7-target genes levels in 22Rv1 cells was determined by qPCR, following vehicle or P5091 treatment (25 μM) for 24 h, and normalized against expression of GAPDH. In C and D the values are the mean +/− SD of three independent experiments.

We treated the 22Rv1 cells with vehicle or various concentrations of P5091 and counted the cells at different times, as indicated in Figure 2A. The P5091 treatment attenuated the proliferation of the 22Rv1 cells in the absence or presence of DHT (Figure 2A). The 22Rv1 cells showed an increase in the number of apoptotic cells upon USP7 inhibitors treatment, as revealed by different assays. The Z-VAD-FMK pan-caspase inhibitor interfered with the P5091-induced citotoxicity in the castration-resistant 22Rv1 prostate cancer cells (Figure 2B); moreover, we observed the activation of the caspase 3 upon P5091 treatment in these cells (Figure 2C).

Interestingly, when the 22Rv1 cells were pretreated with either vehicle or P5091 for 4 hr, followed by addition of cycloheximide (50 μg/ml) to block new protein synthesis, the USP7 inhibitor reduced both the levels of ARFL and ARV7 variant. As final effect, the USP7 inhibitors treatment reduced the levels of mRNA of genes that are specifically regulated by AR full lenght and by AR-V7 isoform (Figure 2D). In the androgen-resistant 22Rv1 cells, the USP7 inhibitor significantly reduced the AR-dependent PSA, PDE9A and FKB5 target genes expression (Figure 2E), as observed in the hormone-sensitive LNCaP cells (Supplementary Figure 1). Additionally, we found that the USP7 inhibitor treatment was able to negatively modulate the mRNA expression of Cdc20, AKT1 and Ube2c, that are considered target genes specific of the AR-V7 variant (Figure 2F). Thus, the USP7 inhibitor treatment is able to negatively modulate the AR-dependent transcription in hormone-sensitive cells and also to downregulate the levels of the ARV7 variant target genes in CRPC cells, suggesting a critical role of USP7 inhibition in CRPC development and maintenance.

### Pharmacological inhibition of USP7 controls CCDC6 stability and impairs the DSBs DNA repair in prostate cancer cells

**Figure 3:**
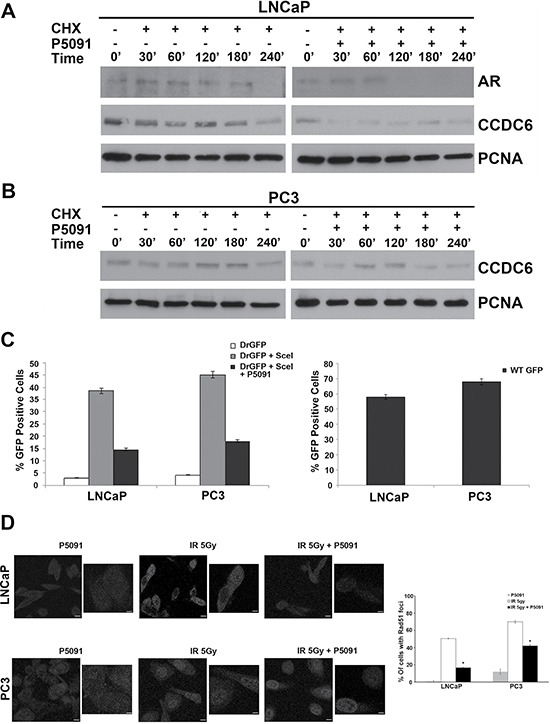
P5091 controls CCDC6 stability and affects the DSBs DNA repair in prostate cancer cells (**A**) LNCaP and (**B**) PC3 prostate cancer cells were pretreated with either vehicle or P5091 (6 μM IC50 in LNCaP and PC3) for 4 h, followed by the addition of cycloheximide (CHX) at 50 μg/ml for the indicated times. Total proteins lysates were subjected to immunoblot analysis using anti-CCDC6 or anti-PCNA antibodies. Anti-AR antibody is also shown in the LNCaP cells. (**C**) LNCaP and PC3 cells were transfected with DR-GFP alone, as control, or together with I-SceI. The percentage of GFP positive cells, compared to controls, have been plotted as histograms that are representative of three independent experiments. Error bars indicate the standard error mean. (**D**) LNCaP and PC3 cells were exposed to 5Gy IR, in presence or not of 5 μM of P5091, followed by 18 h recovery. Immunofluorescence images of the cells stained for Rad51 are shown. Immunofluorescence of LNCaP and PC3 upon P5091 treatment only are also shown. Cells containing more than five foci were scored as positive. The percentage of Rad51 positive nuclei at 18 h from irradiation are shown on the right of the images. Error bars represent standard error mean. Results are representative of at least two independent experiments.

Genetic ablation of USP7 affects the turnover of MDM2 leading to stability of p53, alters the stability of PTEN and p21 and increases the turnover of novel substrates such as the androgen receptor and CCDC6 [8, 14–16]. Appreciable levels of CCDC6 and USP7 proteins have been observed in a series of prostate tumor cell lines independently of the expression of androgen receptor (Figure 1A). Thus, besides the effects of USP7 inhibitors on the stability of AR isoforms and their transcriptional gene targets, we asked whether the treatment with USP7 inhibitor was also able to affect the CCDC6 stability in prostate tumor cells. Hormone-independent PC3 cells and hormone-sensitive LNCaP cells were pretreated with either vehicle or P5091 for 4 hr, followed by addition of cycloheximide (50 μg/ml), in order to block new protein synthesis, for the indicated times. The immunoblot with anti-CCDC6 antibody indicated that the CCDC6 half life was reduced upon the P5091 treatment in these prostate cancer cell lines. The P5091 accelerated the degradation of CCDC6 in PC3 cells and LNCaP versus control cycloheximide alone-treated (Figure 3A and 3B). As expected, the LNCaP cells showed a reduction of the half life of the AR full lenght (AR), upon P5091 treatment (Figure 3A). Then, in order to discern the effect of P5091 directed towards CCDC6 and AR, we investigated the ability to repair the DNA double strand breaks (DSBs) by homologous recombination (HR) in both PC3 and LNCaP cell lines. After transfection of the reporter DR-GFP and the breaking enzyme I-SceI genes, able to induce DSBs, we compared the HDR efficiencies in control or P5091-treated prostate tumor cell lines, by determining the percentage of GFP positive cells by flow cytometry. LNCaP and PC3 cells transfected, or not, with I-SceI plasmid revealed that the treatment with the USP7 inhibitor, accelerating the turnover of CCDC6, yielded significantly lower GFP+ cells, compared to not-treated cells (Figure 3C). In these cells the treatment with USP7 inhibitors, followed by a DNA damage inducer treatment (5Gy IR), produced less Rad51 foci in LNCaP and PC3 cells, than control, suggesting that, upon P5091, the reduced CCDC6 levels impair the Homologous Recombination-directed DNA repair in these cells (Figure 3D).

### The USP7 inhibitor P5091 sensitizes the prostate cancer cells to PARP-inhibitors

Recent studies showed that men with prostate cancer and germline DNA repair aberrations responded to the PARP inhibitor olaparib while patients without DNA repair defects had minimal response [24]. Nevertheless, in few prostate studies the addition of a PARP-1 inhibitor has been also demonstrated to be more effective than hormone therapy alone. Recently, the presence of the ETS gene fusion, in about 50% of prostate cancer patients, has been suggested as a possible predictive biomarker for the response to PARP inhibitors treatment [36–38].

In previous studies, we have reported that low levels of CCDC6 protein sensitize NSCLC and colon carcinoma cells to the PARP inhibitor olaparib [16, 22]. The identification of CCDC6 as a novel USP7 substrate has provided the rationale to establish that the USP7 inhibitor, P5091, by downregulating CCDC6 protein, can modulate the PARP-inhibitors sensitivity in lung neuroendocrine tumors [23]. Thus, we decided to test in prostate cancer cells the sensitivity to PARP inhibitors in combination or not with the USP7 inhibitors.

**Figure 4:**
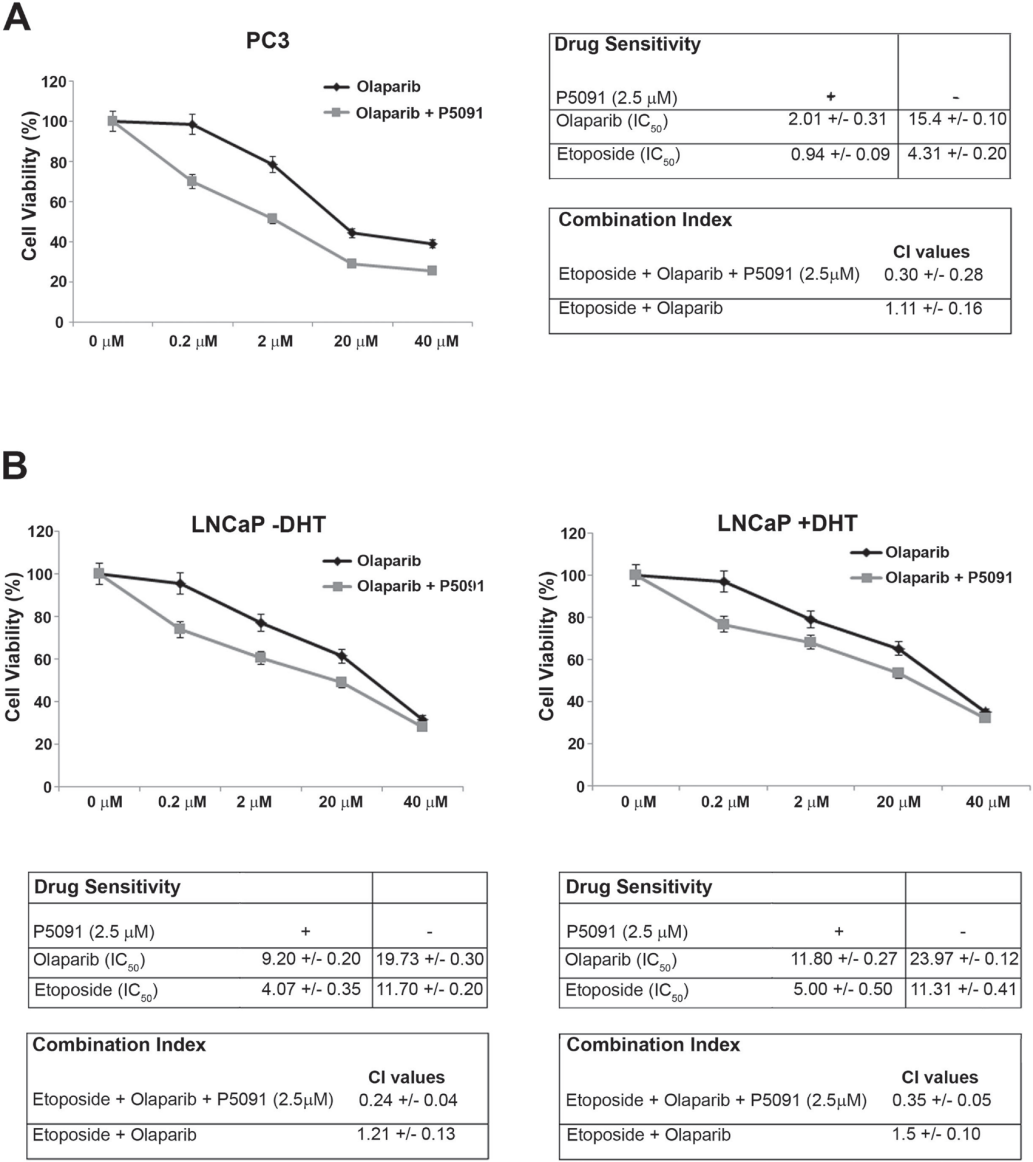
The USP7 inhibitor P5091 sensitizes the prostate cancer cells to PARP-inhibitors (**A**) Left: Surviving fractions of PC3 cells treated, in presence or absence of P5091 (2.5 μM), with olaparib at the indicated doses for 144 h are shown. Right: drugs sensitivity to olaparib and etoposide, in presence or absence of P5091 (2.5 μM) was determined by a modified 3-(4,5-dimethylthiazole-2-yl)-2-5-diphenyltetrazolium bromide assay, CellTiter 96 Aqueous One Solution assay (Promega), as 50% inhibitory concentration (IC50) values. CI according to 1:2 concentration ratio of etoposide and olaparib, in presence or absence of P5091 (2.5 μM), are shown. (**B**) Top: Surviving fractions of LNCaP cells treated, in presence or absence of P5091 (2.5 μM), with olaparib at the indicated doses for 144 h are shown. DHT (10 nM) was added as indicated (− / +). Bottom: drugs sensitivity to olaparib and to etoposide in presence or absence of P5091 (2.5 μM) was determined by a modified 3-(4,5-dimethylthiazole-2-yl)-2-5-diphenyltetrazolium bromide assay, CellTiter 96 Aqueous One Solution assay (Promega), as 50% inhibitory concentration (IC50) values. CI according to 1:2 concentration ratio of etoposide and olaparib, in presence or absence of P5091 (2.5 μM), are shown. CI < 1, CI = 1, CI > 1 indicate synergism, additive effect and antagonism, respectively. In A, on the right, and in B, at the bottom, the values are presented as mean standard deviation of three independent experiments.

In the present study we show that the PARP-inhibitor olaparib is able to induce limited growth inhibition in the hormone-sensitive and in the androgen-independent PC cells. Both cell lines express appreciable levels of CCDC6, but are positive (LNCaP) or negative (PC3) for AR expression (Figures 1A and 3B). The addition of P5091 enhanced the sensitivity to PARP-inhibitor olaparib in PC3 cells [PC3: IC50 = 15.4 μM vs 2.01 μM in presence of 2.5 μM P5091] (Figure 4A) and in the LNCaP cells [IC50 = 19.7 μM vs 9.2 μM, in presence of 2.5 μM P5091, (in absence of DHT); IC50 = 23.9 μM vs 11.8 μM, in presence of 2.5 μM P5091 (in presence of DHT]) (Figure 4B). Moreover, we have monitored cellular apoptosis percentage in LNCaP and PC3 cells, in support of the synergy between USP7 inhibitor and PARP inhibitor (Supplementary Figure 2A–2D). Finally, in both PC3 and LNCaP cells, the combination of olaparib with etoposide showed a synergistic effect in presence of the USP7 inhibitor P5091, [CI < 1], while determined an antagonistic effect, in absence of the USP7 inhibitor [CI > 1] (Figure 4A and 4B). These results suggest that the efficacy of USP7 inhibitors in combination with PARP inhibitors in prostate cancer cells is not dependent on AR expression and may be related to different USP7 targets, including CCDC6.

Notably, by adding back ectopic wt CCDC6 plasmid to the LNCaP treated with P5091, we were able to rescue the weak sensitivity to olaparib, in terms of 50% inhibitor concentration values (IC50) (Supplementary Figure 3), suggesting a pivotal role of CCDC6 as a USP7 substrate. Moreover, in the LNCaP cells, the transient silencing of AR, CCDC6 or both AR and CCDC6 showed that the sensitivity to olaparib is present only in the cells in which occurred the CCDC6 depletion (indipendently from AR depletion) (Supplementary Figure 4A). The effect of AR silencing on PSA transcription have been also reported (in presence or absence of CCDC6) (Supplementary Figure 4B).

**Figure 5:**
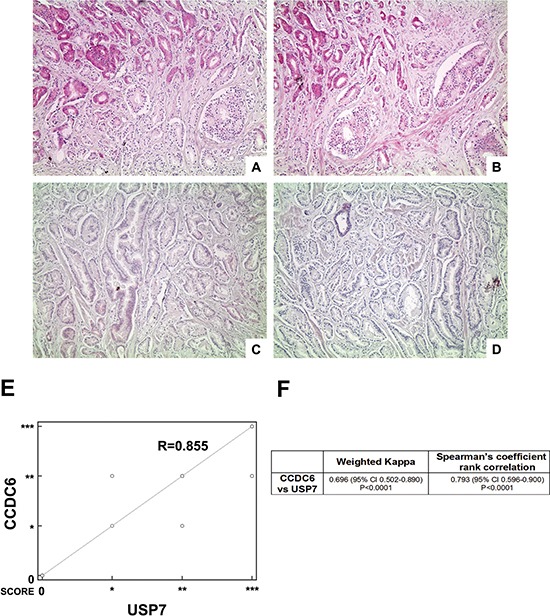
Combined effect of USP7 inhibitors and PARP inhibitors in the 22Rv1 cells (**A**) and (**B**) Top: Surviving fractions of 22Rv1 cells treated, in presence or absence of P5091 (2.5 μM), with olaparib at th indicated doses for 144 h are shown. DHT (10 nM) was added as indicated (−/+). Bottom: Drugs sensitivity to olaparib in presence or absence of P5091 (2.5 μM) was determined by a modified 3-(4,5-dimethylthiazole-2-yl)-2-5-diphenyltetrazolium bromide assay, CellTiter 96 Aqueous One Solution assay (Promega), as 50% inhibitory concentration (IC50) values.

We also tested the combined effect of the USP7 inhibitors and PARP inhibitors in the castration resistant 22Rv1 cells. After treatment with olaparib, the 22Rv1 cells showed a limited growth inhibition. However, the addition of the USP7 inhibitor P5091, at the concentration of 2.5 μM, enhanced the 22Rv1 cells sensitivity to the PARP-inhibitor olaparib [IC50 = 17.9 μM vs 2.9 μM], in presence or absence of DHT (Figure 5A and 5B). The apoptotic percentages also support the synergy between the two inhibitors (Supplementary Figure 2E, 2F). Nevertheless, the immunoblot to detect CCDC6 indicated that the half life of the protein was reduced also in these cells, upon the P5091 pretreatment, beyond ARFL and ARV7 (Figure 2A).

Thus, our data demonstrate that the USP7 inhibitor, P5091, negatively modulates the stability and the transcriptional activity of ARFL and of its V7 truncated variant; moreover, our data indicates that the USP7 inhibitor P5091 reduces levels and function of CCDC6, favouring the sensitivity to PARP inhibitors in hormone-sensitive and most importantly, in castration resistant prostate cancer cells (Figure 4A, 4B).

### In primary prostate tumors the CCDC6 expression levels correlate to USP7 protein levels

**Figure 6:**
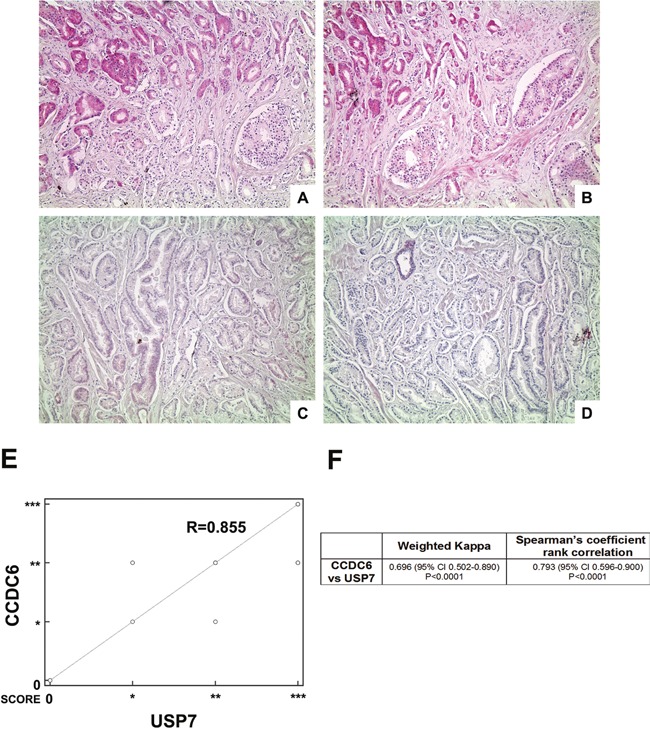
Highly concordant expression of CCDC6 and USP7 (**A**–**D**) Two representative cases of prostate adenocarcinoma (A, B case1; C, D case 2): (A, C) CCDC6 stain (100×), respectively high expression (A) and low expression (C); (B, D) USP7 stain (100×), respectively high expression (B) and low expression (D); (**E**) Scatter plot showing the relationship between USP7 and CCDC6 IHC expression as from prostate adenocarcinoma TMA analysis (correlation coefficient *R* = 0.855); (**F**) The table summarizes the weighted kappa and the Spearman's rank correlation analyses results, both proved to be extremely significant.

For the purpose of assessing CCDC6 expression levels in a heterogeneous group of human prostate tumors, we analysed 28 samples from patients who underwent surgical tumor resection without any previous treatment. The immunostaining showed that expression levels of CCDC6 directly correlated to the protein levels of its deubiquitinating enzyme, USP7 (Figure 6A–6D).

More precisely, TMA immunostaining of CCDC6 expression demonstrated that the protein was barely detectable in 14% of the samples analysed (4 out of 28); a similar pattern of expression was observed for USP7. Nevertheless, the remaining 24 samples analysed displayed a positive immunostaining for CCDC6 and USP7 proteins. A whole list of CCDC6 and USP7 score of intensity with the relative frequencies in the 28 examined TMA cores is resumed in Table 1. Notably, in more than 65% of the samples, the CCDC6 and USP7 levels of expression perfectly matched, disrespectfully of Gleason score (Table 2). Of notice, nearly 50% of the samples exhibited a high intensity of staining of both the proteins. Infact, the correlation analysis between USP7 and CCDC6 IHC expression derived from prostate adenocarcinoma TMA analysis showed a coefficient of *R* = 0.855, as shown by scatter diagram in Figure 6E, reporting CCDC6 expression scores plotted against the score reported for USP7.

**Table 1:** Frequency distribution of the CCDC6/USP7 IHC expression combined scores in the whole series of 28 examined TMA cores

CCDC6/USP7 SCORES	Frequency *n* (%)	GLEASON		
				
0/0	4/28 (14%)	6 (3 + 3)	2/4	19/28 (68%)
		6 (3 + 3)	1/4	
		7 (3 + 4)	1/4	
*/*	1/28 (4%)	7 (4 + 3)	1/1	
**/**	8/28 (29%)	6 (3 + 3)	2/8	
		7 (4 + 3)	4/8	
		7 (3 + 4)	1/8	
		9 (5 + 4)	1/8	
***/***	6/28 (21%)	6 (3 + 3)	1/6	
		7 (3 + 4)	2/6	
		8 (4 + 4)	2/8	
		9 (5 + 4)	1/8	
				
*/**	2/28 (7%)	5 (3 + 2)	1/2	9/28 (32%)
		6 (3 + 3)	1/2	
**/*	2/28 (7%)	6 (3 + 3)	1/2	
		7 (3 + 4)	1/2	
**/***	5/28 (18%)	5 (3 + 2)	1/5	
		7 (4 + 3)	2/5	
		8 (5 + 3)	1/5	
		8 (4 + 4)	1/5	

68% of TMA cores showed a concordant expression of the two proteins, disrespectfully of Gleason score.

**Table 2:** Contingency analysis of CCDC6 or USP7 scores vs clinic-pathological features of the study population (DF: degree of freedom; laterality: monolateral, bilateral)

			Chi-squared	DF	Significance level
**CCDC6**	vs	Gleason	7.291	12	*P* = 0.8378
**USP7**			7.780	12	*P* = 0.8020
**CCDC6**	vs	Stage	0.342	3	*P* = 0.9519
**USP7**			0.804	3	*P* = 0.8485
**CCDC6**	vs	Laterality	0.382	3	*P* = 0.9440
**USP7**			3.891	3	*P* = 0.2734
**CCDC6**	vs	Capsule infiltration	2.956	3	*P* = 0.3985
**USP7**			2.832	3	*P* = 0.4183

The concordance between CCDC6 and USP7 staining values was confirmed by the Cohen's k value (0.696) (Figure 6F) and by the rank correlation analysis, based on 2-tailed non parametric Spearman test, that resulted of statistical significance (*p* < 0.05) (Figure 5F).

## DISCUSSION

Prostate cancer is the most frequently diagnosed tumor in men and the leading cause of cancer-related death followed by cancers of lung and bronchus in US [1]. In the last years the outcome of prostate cancer has been improved because of the evaluation of prostate-specific antigen (PSA) that allows the early detection of asynmptomatic prostate cancer [1]. Nevertheless, for patients with advanced disease with castration resistant prostate cancer characterised by a constitutive activation of AR, depending on AR gene point mutations or truncations, there is an urgent need to develop new treatments [34–37].

Recently, the deubiquitinating enzyme USP7 has been identified as a novel AR co-regulator in prostate cancer cell. USP7 mediates AR deubiquitination and associates with AR, in an androgen dependent manner, driving the transcription of target genes and promoting prostate cells growth [8]. USP7 expression in PC has been correlated with tumor aggressiveness and has been considered as a possible therapeutic target.

We evaluated the efficacy of the USP7 de-ibiquitinase inhibition to affect the growth rate of hormone sensitive and CRPC cells. In this work we report that the USP7 inhibitors negatively modulate the AR-full-length levels and its trascriptional abilities and proliferative effects in PC cells. Moreover, the pharmacological inhibition of the de-ubiquitinase USP7 also affects the growth of castration resistance prostate cancer cells, 22Rv1, that express both functional AR full lenght and its constitutive variant isoform V7, reducing the levels of the variant V7, possible driven by the ability to heterodimerize with the full lenght androgen receptor [39]. Upon USP7 inhibition the expression of genes regulated by the full-length AR, such as PSA and FKBP5 [40–42] is reduced. Importantly, genes distinctly regulated by the AR-V7, such as UBE2C and CDC20, also are inhibited by USP7 inhibition in 22Rv1 cells, whereas bicalutamide had no effect [43]. Interestingly, the ubiquitin-conjugating enzyme 2C (UBE2C) is aberrantly increased in many cancer types and is reported to be casually involved in prostate cancer development and progression [44]. Moreover, its partner cdc20 has been involved in prostate tumorigenesis by increasing chromosome instability [45] and has received more interest as a promising target for novel therapeutic strategy by small molecule inhibitors [46].

Recently, it has been reported that PARPi could represent a new therapeutic option for a large percentage of patients with CRPC harboring DNA repair gene mutations [24]. Almost 30% of patients with CRPC have these alterations and they have a high possibility to respond to treatment [47]. Extending the use of PARP inhibitors, beyond tumors with defective BRCA1/2, ATM, CHEK2, Fanconi's Anemia genes is of great interest, expecially in prostate cancer, where mutations in DNA repair genes are rare [24].

The use of olaparib as a new therapeutic strategy for CRPC, tailored on the basis of the genomic alterations of the tumor, is limited by the absence of standard test in the clinical practice. Thus, besides the production of specific probes to identify specific gene alterations, alternative strategies are required in order to generate biological data that can drive the best therapeutic choice expecially in the castration resistant prostate cancer (CRPC).

USP7, besides AR, has different substrates including PTEN [15] and CCDC6, a tumor suppressor protein whose deficiency affects DNA repair mechanism by homologous recombination and sensitizes tumor cells to PARP inhibitors treatment [16].

Recently, we have reported that low levels of CCDC6 associates with defects in DNA repair by Homologous Recombination (HR) affecting cells behaviour and cells sensitivity to PARP inhibitors treatment in lung and colon cancer models [16, 22]. Moreover, we have observed a lethal effect combining the inhibitors of USP7 and PARP enzymes in lung neuroendocrine tumor expressing USP7 and CCDC6 proteins [23].

In this work we also report that the pharmacological inhibition of USP7 leads to downregulation of CCDC6 protein and results in DNA repair defect that sensitize the androgen-independent, hormone-sensitive and CRPC cells to PARP inhibitor treatment, alone or in combination with standard radio- and chemotherapies.

The inhibition of PARP enzymes as anticancer strategy has been established on the basis of the biological concept of synthetic lethality, for which two genomic events, that are each relatively innocuous individually, become lethal when occurring together. When PARP enzymes are pharmacologically inhibited, the DNA single strand breaks cannot be repaired and eventually progress to toxic double strand breaks (DSBs), that result to be lethal in cells that lack HR repair capacity or have lost DNA repair genes [48–49].

Therefore, in prostate cancer cells with DNA repair defects or PTEN homozygous deletion, previous literature had suggested radiosensitization from PARP inhibition, accordingly to the concept of “native synthetic lethality” [48–49]. Here we suggest that the lethal effect obtained by the combined treatment of the PARP inhibitors with the USP7 inhibitors in prostate cancer cells that express CCDC6, and its de-ubiquitinating enzyme USP7, may be considered an “induced synthetic lethality”.

In this work we have detected appreciable levels of CCDC6 and USP7 proteins, that in primary tumors perfectly matched in more than 65% of samples, disrespectfully of Gleason score. Nevertheless, it is interesting to observe that in the patient samples affected by a high gleason score (8–9), USP7 and CCDC6 showed the highest score of intensity (+++/+++) (Table 1). These findings need further investigations.

Thus, the USP7 inhibitors can offer the chance to modulate the growth ability of prostate cancer cells: on one side they can, by downregulating the levels of both isoforms, negatively modulate the AR-FL and the ARV7 dependent proliferative and trascriptional abilities; on the other side by downregulating CCDC6, they can affect the homologous directed DNA repair and sensitize the castration resistant cancer cells to the PARP inhibitors treatment.

We believe that it is mandatory to extend the analysis of CCDC6 and USP7 protein expression to a larger series of prostate tumor samples in order to strenghten our observations about the joined/combined expression of the two proteins, and to predict the outcome of this tumor following the treatment with the USP7 inhibitors and PARP inhibitors, in combination with genotoxic durgs. Moreover, we gathered gene-expression datasets from prostate adenocarcinoma, from TCGA via Cbioportal. Our analysis revealed a strong correlation between CCDC6 and USP7 mRNA expression levels across a series of studies analyzed [50–54]. Correlation values between CCDC6 and AR, and USP7 and AR were mostly equivalent in all the studies. Additionally, a correlation analysis between CCDC6 or USP7 and AR substrates (KLK3, CDC20, UBE2C, AKT1) proved to be consistent across the studies queried for gene expression data. A Table has been added as supplementary material (Supplementary Table 2).

Finally, we propose that the assessment of CCDC6 and USP7 tissue expression could provide us with a predictive tool to manage prostate cancer patients at advanced stage. The inclusion of AR evaluation should be considered, as suggested by the staining of few representative prostate cancer samples (Supplementary Figure 5). Nevertheless, in order to improve the sensitivity and specificity of the IHC test for USP7 and CCDC6, a wider panel of biomarkers should be envisaged, also including UBE2C and CDC20, beyond the detection of PSA serum levels.

## MATERIALS AND METHODS

### Cell lines, drugs and chemicals

The PC3, LNCaP and the 22Rv1 prostate cancer cell lines were obtained from the American Type Culture Collection (Rockville, MD, USA). The PC3 and the LNCaP were cultured in RPMI 1640 (Gibco, Paisley, UK), supplemented with 10% fetal bovine serum (Gibco, Paisley, UK) and 1% penicillin/streptomycin (Gibco, Paisley, UK). The 22Rv1 cell lines were cutured in RPMI 1640 (Gibco, Paisley, UK), supplemented with 20% fetal bovine serum (Gibco, Paisley, UK) and 1% penicillin/streptomycin (Gibco, Paisley, UK). Olaparib (AZD2281) and P005091 were provided by SelleckChem. The androgen 5a-dihydrotestosterone (DHT), the cycloheximide and etoposide were obtained from SIGMA-Aldrich, Inc. The caspase-3 inhibitor Z-VAD-FMK was from Merck Millipore Corporation.

### Sensitivity test and design for drug combination

Antiproliferative activity was determined by a modified 3-(4,5-dimethylthiazole-2-yl)-2-5- diphenyltetrazolium bromide assay, CellTiter 96 AQueous One Solution assay (Promega), as 50% inhibitory concentration (IC50) values. Briefly, cells were plated in quintuplicate in 96-well plates at a density of 1000 cells per well, and continuously exposed to each drug for 72h. Each assay was performed in quintuplicate and IC50 values were expressed as mean +/− standard deviation. The results of the combined treatment were analyzed according to the method of Chou and Talaly by using the CalcuSyn software program [55]. The resulting combination index (CI) is a quantitative measure of the degree of interaction between different drugs. A CI value of unity denotes additive activity while CI > 1 denotes antagonism, and CI < 1 denotes synergy between agents.

### Protein extract and western blot analysis

Total cell extracts (TCE) were prepared with lysis buffer (50 mM Tris–HCl pH 7.5, 150 mM NaCl, 1% Triton X-100, 0.5% Na Deoxycholate, 0.1% SDS) and a mix of protease inhibitors. Protein concentration was estimated by a modified Bradford assay (Bio-Rad). For Western blotting, cell lysates were separated by SDS-PAGE (10% polyacrylamide) and the proteins were transferred to a PVDF membrane. Membranes were blocked with 5% TBS-BSA and incubated with the primary antibodies. Immunoblotting experiments were carried out according to standard procedures and visualized using the ECL chemiluminescence system (Amersham/Pharmacia Biotech). As a control for equal loading of protein lysates, the blotted proteins were probed with antibody against anti-γ-tubulin protein.

### Reagents and antibodies

For biochemical analysis the antibodies anti-CCDC6 (ab56353) Abcam, the anti-USP7 (A300-033A) Bethyl, anti-AR (sc-7305) Santa Cruz Biotechnology (CA, USA) and anti-γ-tubulin (T6557), SIGMA-Aldrich, Inc, were utilized. Secondary antibodies were from Biorad, California. For the immunohistochemical studies the antibodies anti-CCDC6, [(HPA-019051), Sigma-Aldrich, Co. LLC] and anti-USP7 [(HPA-015641), Sigma-Aldrich, Co. LLC] were utilized. The anti-AR (sc-7305) was from SCBT (CA, USA).

### Apoptosis assays

PC3, LNCaP and 22Rv1 cells were treated with P5091 at 12.5 μM for 24 hours and apoptosis was quantified by measuring Caspase 3/7 activation using the Caspase-Glo 3/7 assay (Promega) according to the manufacturer's instructions.

### TMA and IHC

Archival tumor samples from 28 patients (smokers and nonsmokers) with prostate cancer were retrieved from the files of the Pathology Section of the Departement of Advanced Biomedical Sciences, University Federico II of Naples, with informed consent and standard IRB approvals. Clinicopathologic data were recorded. The patients’ age ranged between 46 and 73 years, with a mean of 63.8 years, median age 64 years. Patients underwent surgery between 2003 and 2005. After surgical resection, tissues were fixed in 10% neutral buffered formalin and embedded in paraffin blocks. Sections (4 μm thick) were stained with haematoxylin and eosin (H&E). Histologic grading and pathological staging were performed according to WHO guidelines [prostate book]. The pathologic analysis was done in a blinded manner with respect to the patients’ clinical data. Tissue microarray (TMA) was built using the most representative areas from each single case. Tissue cores with a diameter of 3 mm were punched from morphologically representative tissue areas of each ‘donor’ tissue block and brought into one recipient paraffin block (3 × 2.5 cm) using a manual tissue arrayer, as described [56]. The same TMA was used for both CCDC6 and USP7 staining. Immunohistochemistry was performed as described [16, 23]. The immunohistochemical staining of CCDC6 and USP7 was evaluated semiquantitatively as the percentage of positive cells (with either nuclear or cytoplasmic localization). Cells were classified as follow: 0 (< 5%); + (5–25%); ++ (26–50%) and +++ (> 50%).

### Statistical analysis

Statistical analysis was performed with SPSS package for Windows (IBM Corp. Released 2013. IBM SPSS Statistics for Windows, Version 22.0. Armonk, NY: IBM Corp.). Statistical differences were determined by two-tailed Student's *t* test. Statistical significance is dyplayed as: *p < 0.05; **p < 0.01, and ***p < 0.001. The χ^2^ test was used to compare the quantitative differences of CCDC6 or USP7 staining and the clinic-pathological features of the study population. The p-value was considered significant if < 0.05. To determine the index between the immunohistochemical staining scores of CCDC6 and USP7, the Cohen's weighted kappa statistic was calculated. Chance-corrected agreement was considered poor if K < 0.00, slight if K was between 0 and 0.20, fair if K was between 0.21 and 0.40, moderate if K was between 0.41 and 0.60, substantial if K was between 0.61 and 0.80, and almost perfect if K was > 0.80. Nonparametric Spearman rank correlation test was performed and the p-value was considered significant if < 0.05.

### Real time PCR

PCR reactions were performed on RNA isolated from cell lines using RNeasy Mini Kit (Qiagen) and reverse-transcribed using MuLV RT (Invitrogen). qRT-PCR was performed with Syber Green (Agilent) using primers as listed in Supplementary Table 1. To calculate the relative expression levels we used the 2−ΔΔCT method.

### Immunofluorescence staining

After treatment with the USP7 inhibitor P5091, the PARP inhibitor olaparib or with both the drugs, as indicated, the PC3, LNCaP and 22Rv1 cells were fixed with 4% paraformaldehyde and treated with phosphate-buffered saline (PBS)/0.25% Triton X-100. After staining with primary antibody, the recombinase Rad51, cells were washed in PBS and incubated for 30 min at room temperature with the secondary antibody. Nuclei were visualized by staining with DAPI. Cells with a number of Foci > 6 were scored as positive.

### Plasmids and transfection

PcDNA4ToA-CCDC6 plasmids were transfected in LNCaP cells with FuGene HD (Promega) and have been described elsewhere [57]. The DR-GFP reporter plasmid is based on a construct developed by M. Jasin [58] and contains two mutated GFP genes. CCDC6 shRNA (pLKO.1 puro) was from Sigma-Aldrich. For transient CCDC6 silencing, LNCaP cells were transfected with a plasmid pool (shCCDC6, NM_005436) or a pool of nontargeting vectors (sh control) by Fugene (Promega) for 48 hours. In LNCaP cells the transient AR silencing was obtained by AR siRNA (h: sc29204, Santa Cruz Biotechnology, Inc, USA).

## SUPPLEMENTARY MATERIALS FIGURES AND TABLES


