# Supplementary material for: The combined effect of USP7 inhibitors and PARP inhibitors in hormone-sensitive and castration-resistant prostate cancer cells
Source: Oncotarget. 2017 Mar 22;8(19):31815–29. doi: 10.18632/oncotarget.16463 (PMC5458250; doi:10.18632/oncotarget.16463)
Supplement: Supplementary file 1 [file oncotarget-08-31815-s001.pdf]

The combined effect of USP7 inhibitors and PARP inhibitors in hormone-sensitive and castration-resistant prostate cancer cells

Supplementary Materials

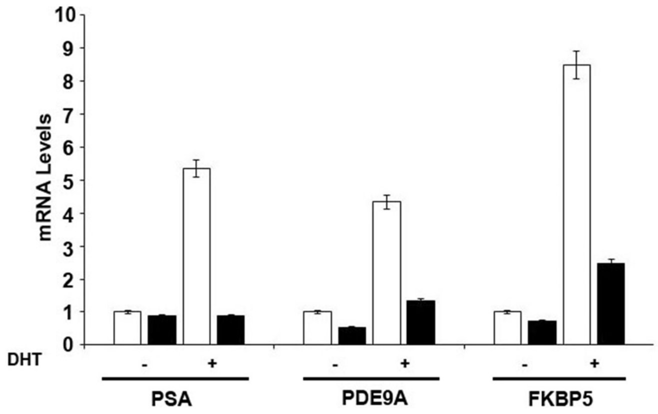

Supplementary Figure 1: P5091 treatment affects AR-target gene levels. Expression of AR-target gene levels in LNCaP cells was determined by qPCR, following vehicle or P5091 treatment (25 µM) for 24 h, and normalized against expression of GAPDH. The values are the mean +/− SD of three independent experiments.

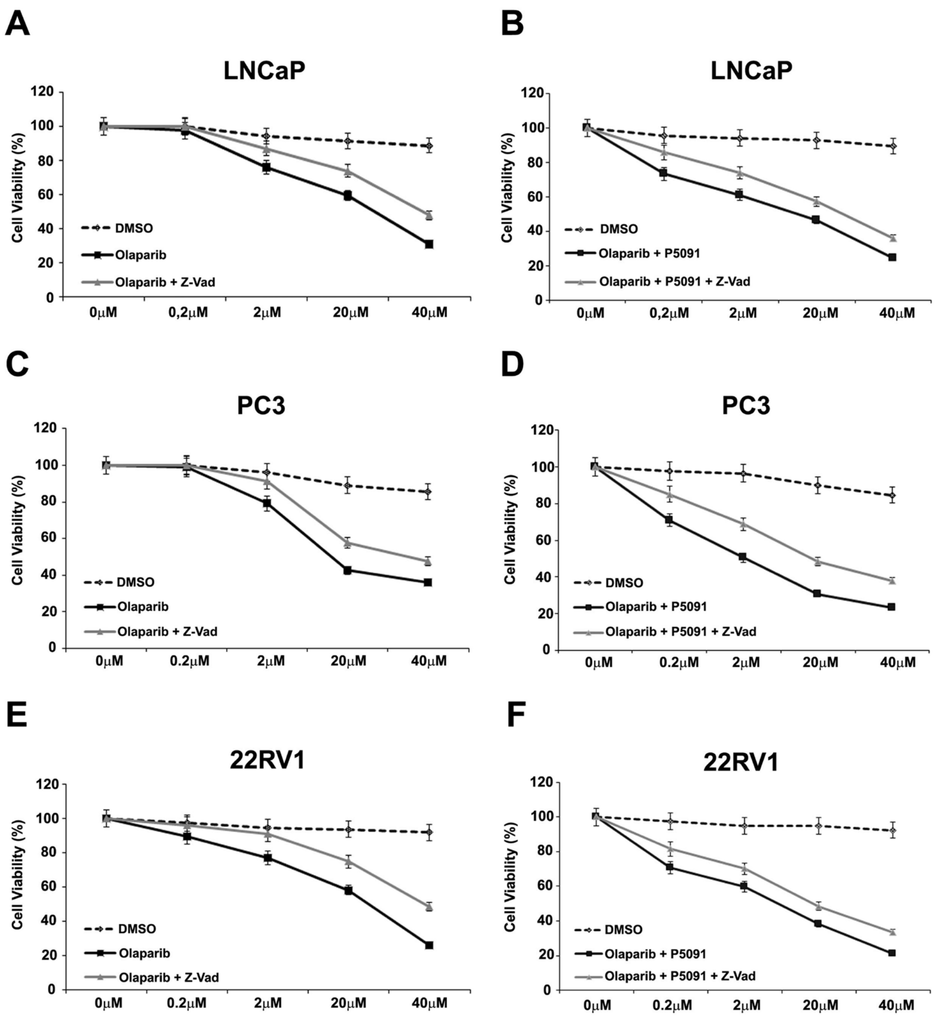

Supplementary Figure 2: The addition of P5091 to olaparib shows dose-dependent cytotoxic effects in prostate cancer cell lines. LNCaP, PC3 and 22Rv1 cells were seeded in 96-well plates and 24 h later exposed to olaparib at the indicated doses, together with P5091 (at a fixed dose of 2.5 μM) in presence or absence of the pan-caspase inhibitor Z-VAD-FMK (20 μM) for 72 h, and analysed for viability by using a modified 3-(4,5-dimethylthiazole-2-yl)-2-5-diphenyltetrazolium bromide assay, CellTiter 96 Aqueous One Solution assay (Promega), as 50% inhibitory concentration (IC50) values. The values are presented as mean standard deviation of three independent experiments. Surviving fraction of LNCaP, PC3 and 22Rv1 cells are shown, compared to olaparib treated cells.

Drug Sensitivity

Cell line	Olaparib P5091 2.5μM	Olaparib
LNCaP^{EV}	8.00 +/- 0.12	20.37 +/- 0.60
LNCaP^{CCDC6+}	13.40 +/- 0.31	20.80 +/- 0.43

Supplementary Figure 3: CCDC6 reconstitution affects the olaparib sensitivity. cells were treated, in presence or absence of P5091 (2.5 μM), with olaparib at th indicated doses for 144 h. In the LNCaP transfected with CCDC6 (LNCaP^{CCDC6+}) or with the empty vector (LNCaP^{EV}) the drugs sensitivity to olaparib, in presence or absence of P5091 (2.5 μM), was determined by a modified 3-(4,5-dimethylthiazole-2-yl)-2-5-diphenyltetrazolium bromide assay, CellTiter 96 Aqueous One Solution assay (Promega), as 50% inhibitory concentration (IC50) values.

A

Drug Sensitivity	
**Cell Line**	**Olaparib (μmol/L)**
**LNCaP Sh CTRL**	**18.05 +/- 0.45**
**LNCaP Sh CCDC6**	**6.67 +/- 0.86**
**LNCaP SiRNA AR**	**18.60 +/- 0.48**
**LNCaP Sh CCDC6 + SiRNA AR**	**6.20 +/- 0.48**

B

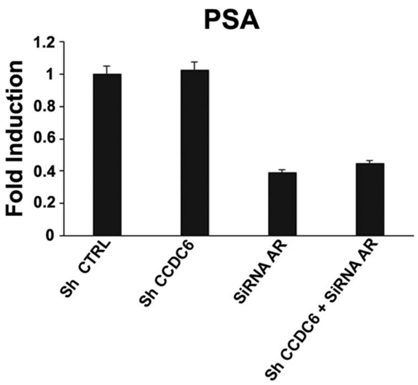

C

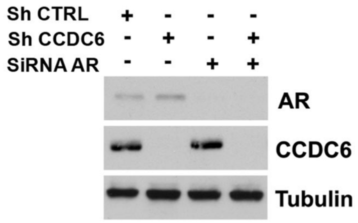

Supplementary Figure 4: Transient silencing of AR, CCDC6 or both AR and CCDC6 in LNCaP cells. (**A**) Drugs sensitivity to olaparib in LNCaP cells transiently silenced for CCDC6, AR or both CCDC6 and AR was determined by a a modified 3-(4,5-dimethylthiazole-2-yl)-2-5-diphenyltetrazolium bromide assay, CellTiter 96 Aqueous One Solution assay (Promega), as 50% inhibitory concentration (IC50) values. (**B**) Expression levels of the AR-target gene PSA was determined, by qPCR, in LNCaP cells following transient silencing of CCDC6, AR, CCDC6 and AR, or control and normalized against expression of GAPDH. The values are the mean +/− SD of three independent experiments. (**C**) LNCaP cells were transfected with control shRNAs (shCTRL), sh-CCDC6, siRNA AR, or shCCDC6 + siRNA AR. The CCDC6 and the AR protein depletion were assessed by Western Blot. Antitubulin immunoblot is shown as loading control.

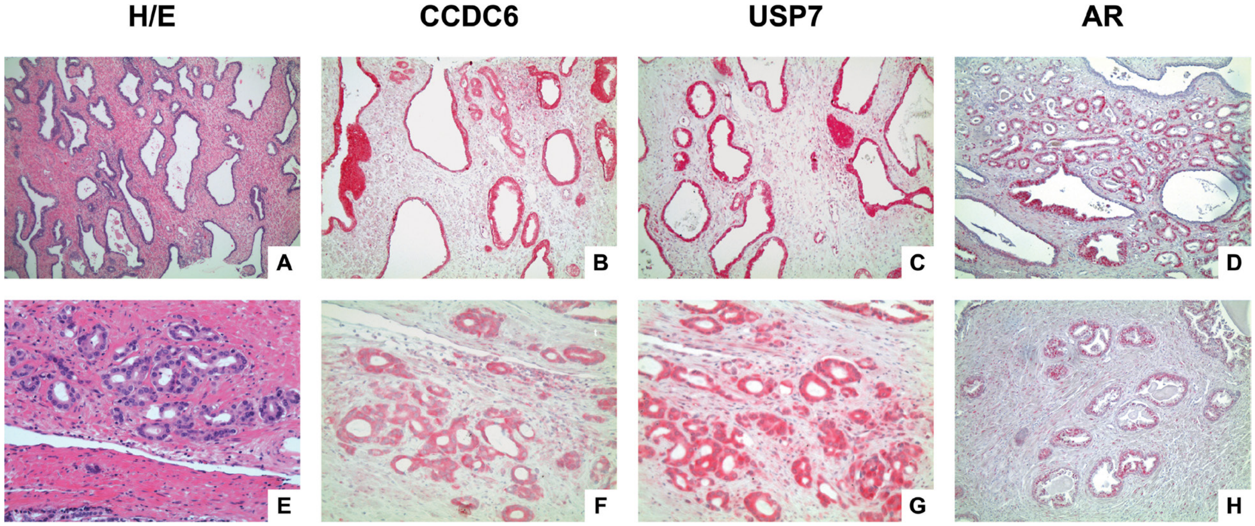

Supplementary Figure 5: AR immunostaining in primary prostate adenocarcinoma. The figure shows two representative cases of prostate adenocarcinoma (case #1**A**–**D**,; case #2**E**–**H**) stained with anti-CCDC6 (B, F) anti-USP7 (C, G) and anti-AR (D, H), A,E: hematoxylin/eosin staining. (A, B, C, D: magnification 100×; E, F, G, H: magnification 200×).

Supplementary Table 1: Oligi sequence of the utilized primers

Target	Forward primer (5′-3′)	Reverse primer (3′-5′)
**PSA**	TGTGTGCTGGACGCTGGA	CACTGCCCCATGACGTGAT
**PDE9A**	GATCCCAATGTTTGAAACAGTGAC	TCCCAAAGTGGCTGCAGC
**FKBP5**	GCGGAGAGTGACGGAGTC	TGGGGCTTTCTTCATTGTTC
**CDC20**	CGGAAGACCTGCCGTTACATTC	CAGAGCTTGCACTCCACAGGTA
**AKT1**	TGGACTACCTGCACTCGGAGAA	GTGCCGCAAAAGGTCTTCATGG
**UBE2C**	TGGTCTGCCCTGTATGATGT	AAAAGCTGTGGGGTTTTTCC

Supplementary Table 2: Table shows Pearson and Spearman values for each correlation test between *CCDC6, USP7, AR, KLK3, UBE2C, CDC20 and AKT1* mRNA levels, as resulted in five studies included in TCGA Cancer Genome Atlas and queried through the cbioportal software

	**Prostate adenocarcinoma (Broad/Cornell, Nat. Genet. 2012, 44, 685–9.)**		**Prostate Adenocarcinoma (MSKCC, Cancer Cell 2010, 18, 11–22)**		**Metastatic Prostate Cancer, SU2C/PCF Dream Team (Robinson et al., Cell 2015, 161, 1215–1228).**		**Prostate Adenocarcinoma (TCGA, Provisional).**		**Prostate Adenocarcinoma (TCGA, Cell 2015, 163, 1011–1025)**	
	Pearson	*Spearman*	*Pearson*	*Spearman*	*Pearson*	*Spearman*	*Pearson*	*Spearman*	*Pearson*	*Spearman*
**CCDC6** vs **USP7**	0.739	0.735	0.728	0.658	0.324	0.387	0.430	0.462	0.471	0.529
**CCDC6** vs AR	NA	NA	0.169	0.263	0.252	0.285	0.352	0.388	0.324	0.419
**CCDC6** vs KLK3	−0.450	−0.286	0.176	0.209	0.135	0.172	−0.193	−0.172	−0.112	−0.068
**CCDC6** vs UBE2C	0.579	0.538	−0.247	−0.236	−0.003	0.025	0.133	0.048	0.009	0.022
**CCDC6** vs CDC20	0.631	0.379	−0.494	−0.459	0.001	0.044	0.071	0.099	0.080	0.100
**CCDC6** vs AKT1	−0.019	0.011	0.217	0.152	0.153	0.254	−0.190	−0.202	−0.216	−0.252
**USP7** vs AR	NA	NA	0.324	0.397	0.293	0.322	0.493	0.559	0.415	0.462
**USP7** vs KLK3	−0.275	−0.301	0.169	0.221	0.181	0.224	−0.275	−0.248	−0.204	−0.186
**USP7** vs UBE2C	0.360	0.320	−0.304	−0.370	0.032	0.112	0.128	0.137	0.109	0.143
**USP7** vs CDC20	0.216	0.071	−0.640	−0.609	0.017	0.068	0.160	0.152	0.196	0.169
**USP7** vs AKT1	0.020	0.030	0.210	0.147	0.432	0.493	0.091	0.069	0.015	−0.025
	mRNA Expression z-Scores (microarray)		mRNA Expression Z-Scores vs Normals		mRNA Expression z-Scores (RNA Seq RPKM)		mRNA Expression z-Scores (RNA Seq V2 RSEM)		mRNA Expression z-Scores (RNA Seq V2 RSEM)	
**Samples**	112		216		150		499		333	
